# Supplementary figures and images for: Effectiveness of COVID-19 shelter-in-place orders varied by state
Source: PLoS One. 2020 Dec 31;15(12):e0245008. doi: 10.1371/journal.pone.0245008 (PMC7775080; doi:10.1371/journal.pone.0245008)

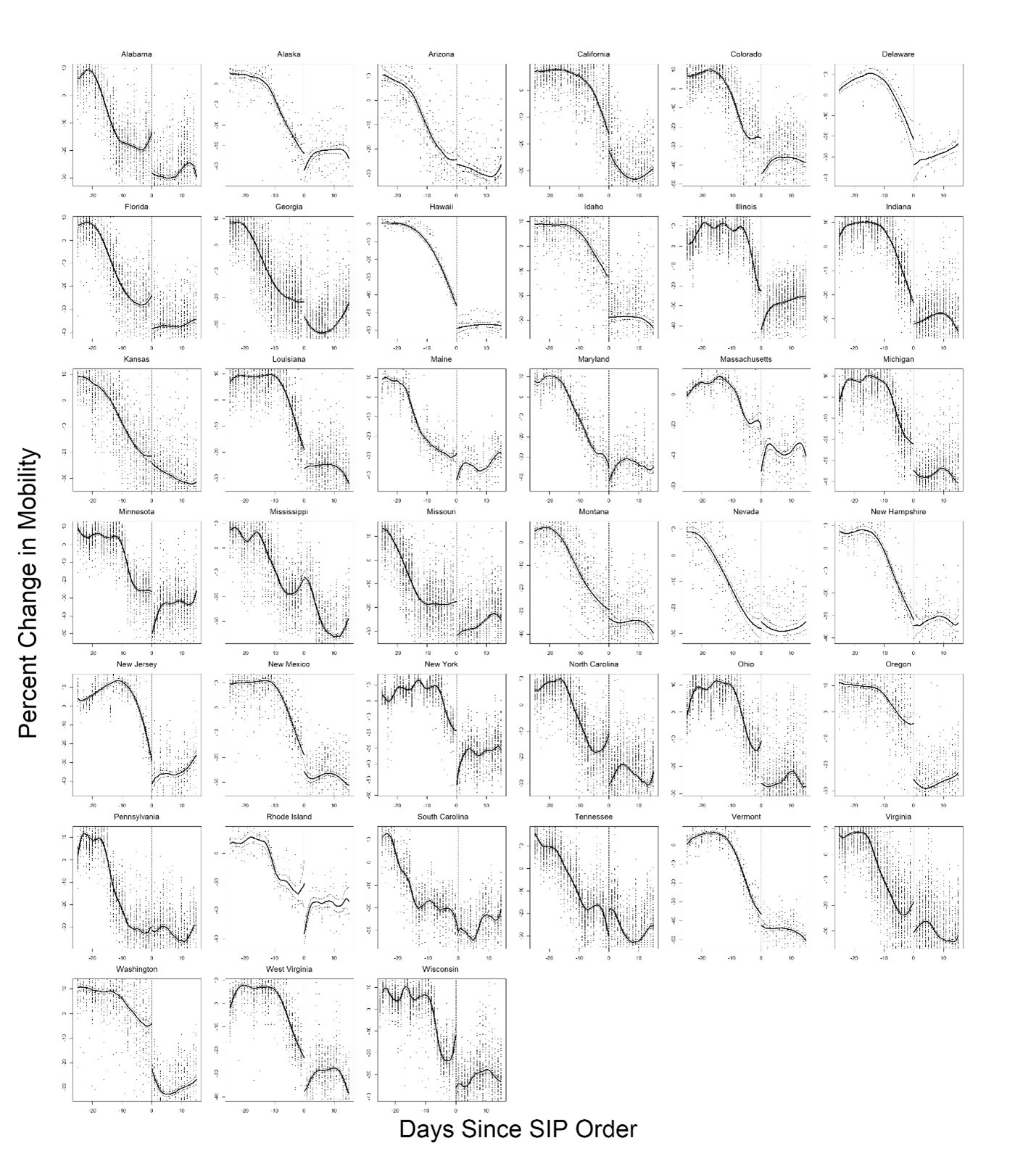

Supplement: S1 Fig — State-specific RD plots using the main analytic approach described in the manuscript. (TIF) [file pone.0245008.s001.tif]

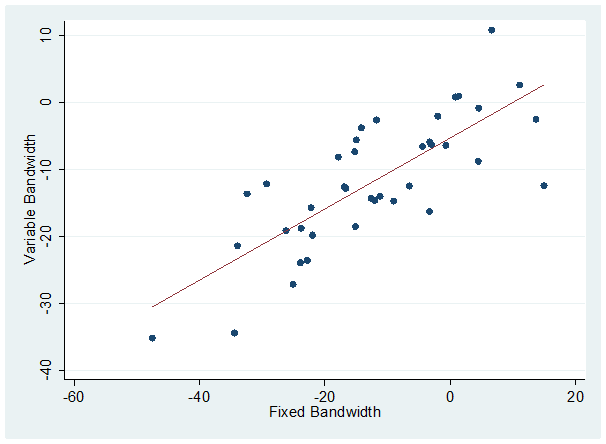

Supplement: S2 Fig — These estimates are generated using the approach described in the manuscript. The variable bandwidth allows the bandwidth to vary by state. The fixed bandwidth uses a single bandwidth of 2.9 for all states. This bandwidth is taken from the primary national specification. The correlation between the two is 0.77. (TIF) [file pone.0245008.s002.tif]

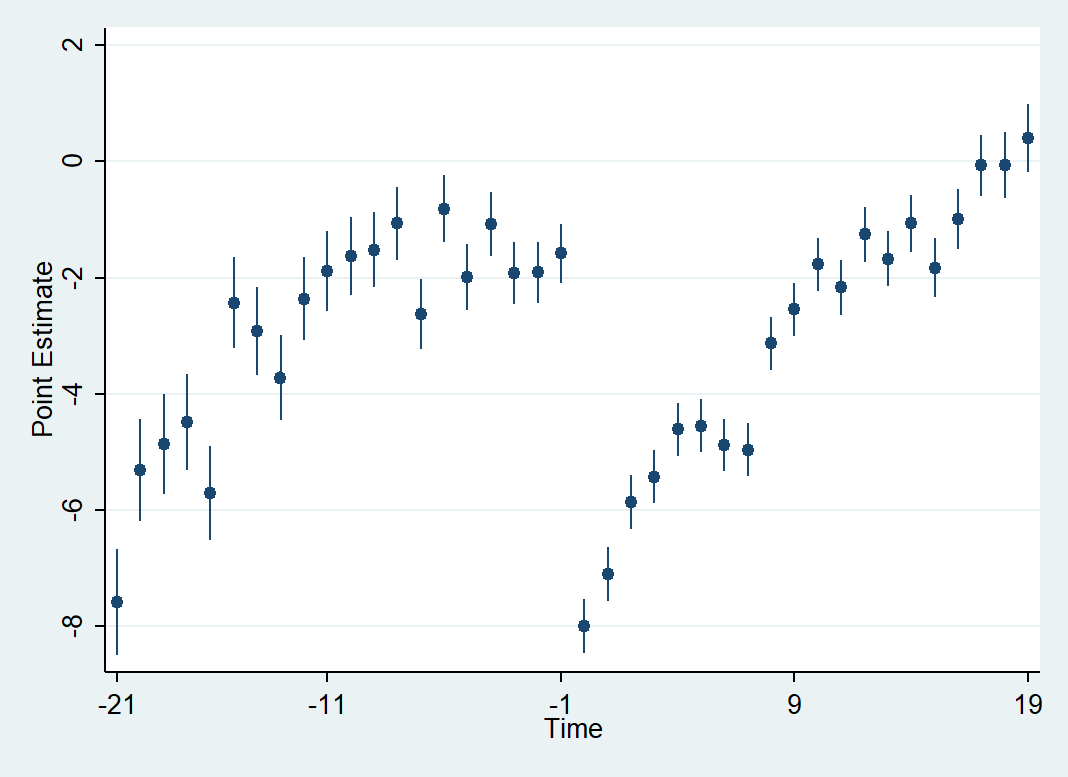

Supplement: S3 Fig — These estimates are from an event-study specification, with the x-axis reflecting days relative to SIP order enactment. The specification includes date and county fixed effects. Leads and lags are censored at +/- 21, and estimates/CIs are accumulated to that point. The outcome is mean mobility, and the coefficient of interest is on the day after SIP enactment. (TIF) [file pone.0245008.s003.tif]
