# Supplementary material for: Effectiveness of COVID-19 shelter-in-place orders varied by state
Source: PLoS One. 2020 Dec 31;15(12):e0245008. doi: 10.1371/journal.pone.0245008 (PMC7775080; doi:10.1371/journal.pone.0245008)
Supplement: S1 Table — This presents mean state-level coefficients and confidence intervals for each mobility category. RD methods are as described in the manuscript. (DOCX) [file pone.0245008.s004.docx]

**S1 Table:** RD estimate by mobility category

| **Category** | **Mean Estimate** | **Mean 95% Lower CI** | **Mean 95% Upper CI** |
| --- | --- | --- | --- |
| Overall (N=17,682) | -11.8 | -17.5 | -6.1 |
| Recreation (N=16,073) | -8.8 | -14.6 | -3.1 |
| Grocery Stores (N=12,639) | -13.7 | -20.2 | -7.2 |
| Parks (N=5,543) | -18.5 | -56.3 | 19.9 |
| Transit (N=11,211) | -4.2 | -16.9 | 8.5 |
| Workplace (N=13,093) | -8.1 | -12.5 | -3.8 |

This presents mean state-level coefficients and confidence intervals for each mobility category. “N” indicates the total number of observations used around the threshold. RD methods are as described in the manuscript.
