# Supplementary material for: Effectiveness of COVID-19 shelter-in-place orders varied by state
Source: PLoS One. 2020 Dec 31;15(12):e0245008. doi: 10.1371/journal.pone.0245008 (PMC7775080; doi:10.1371/journal.pone.0245008)
Supplement: S2 Table — This is a state-level correlation matrix of RD estimates of the effect of SIP orders on each category of mobility. There is low to high correlation across all estimates. Changes in workplace mobility appears to be negatively correlated with changes in mobility for all other categories. (DOCX) [file pone.0245008.s005.docx]

**S2 Table.** **Correlation matrix of category-specific RD estimates.**

|  | **Index** | **Recreation** | **Grocery Stores** | **Parks** | **Transit** | **Workplace** |
| --- | --- | --- | --- | --- | --- | --- |
| **Index** | 1 |  |  |  |  |  |
| **Recreation** | 0.52 | 1 |  |  |  |  |
| **Grocery Stores** | 0.63 | 0.77 | 1 |  |  |  |
| **Parks** | 0.71 | 0.44 | 0.35 | 1 |  |  |
| **Transit** | 0.46 | 0.75 | 0.49 | 0.39 | 1 |  |
| **Workplace** | -0.01 | -0.59 | -0.32 | -0.10 | -0.45 | 1 |

This is a state-level correlation matrix of RD estimates of the effect of SIP orders on each category of mobility. There is low to high correlation across all estimates. Changes in workplace mobility appears to be negatively correlated with changes in mobility for all other categories.
