# Supplementary material for: Effectiveness of COVID-19 shelter-in-place orders varied by state
Source: PLoS One. 2020 Dec 31;15(12):e0245008. doi: 10.1371/journal.pone.0245008 (PMC7775080; doi:10.1371/journal.pone.0245008)
Supplement: S3 Table — These are state-level, category-specific bivariable results regressing coefficients on the socioeconomic and epidemiological factors. Coefficient estimates for continuous variables represent the estimated effect of one standard deviation change in the predictor. A positive coefficient represents a smaller reduction in mobility. N = 39. Robust standard errors are used. (DOCX) [file pone.0245008.s006.docx]

**S3 Table:** Mobility category-specific bivariable results

|  | **Recreation** | **Grocery Stores** | **Parks** | **Transit** | **Workplace** |
| --- | --- | --- | --- | --- | --- |
| **Trump Vote Share** | 2.45 | 2.22 | 20.82 | 3.45 | -0.07 |
| 90% CI Lower | -0.75 | -2.48 | 5.87 | 0.55 | -2.56 |
| 90% CI Upper | 5.66 | 6.91 | 35.78 | 6.36 | 2.43 |
|  |  |  |  |  |  |
| **Pop Density** | -0.08 | -5.57 | -13.46 | -0.51 | 0.34 |
| 90% CI Lower | -0.80 | -6.39 | -16.36 | -1.11 | -0.12 |
| 90% CI Upper | 0.64 | -4.75 | -10.56 | 0.10 | 0.79 |
|  |  |  |  |  |  |
| **Early SIP** | 4.61 | 3.01 | -33.62 | 3.13 | -7.54 |
| 90% CI Lower | -7.01 | -9.77 | -71.45 | -9.06 | -14.79 |
| 90% CI Upper | 16.22 | 15.80 | 4.20 | 15.32 | -0.29 |
|  |  |  |  |  |  |
| **Per Capita Income** | -2.23 | -4.65 | -17.37 | -3.08 | 1.24 |
| 90% CI Lower | -5.27 | -8.78 | -25.57 | -5.78 | -1.33 |
| 90% CI Upper | 0.81 | -0.53 | -9.18 | -0.38 | 3.80 |
|  |  |  |  |  |  |
| **Poverty Rate** | 4.28 | 3.23 | 8.27 | 4.06 | -1.51 |
| 90% CI Lower | 1.05 | -1.11 | -3.19 | 1.24 | -4.17 |
| 90% CI Upper | 7.51 | 7.56 | 19.73 | 6.87 | 1.14 |
|  |  |  |  |  |  |
| **% Black** | 2.22 | 0.91 | 0.66 | 3.33 | -0.29 |
| 90% CI Lower | -1.87 | -4.20 | -9.84 | -0.55 | -3.11 |
| 90% CI Upper | 6.32 | 6.03 | 11.17 | 7.21 | 2.53 |
|  |  |  |  |  |  |
| **Confirmed Cases** | 2.21 | -1.11 | -2.37 | 2.52 | 4.15 |
| 90% CI Lower | -5.15 | -11.74 | -40.96 | -3.70 | -2.56 |
| 90% CI Upper | 9.58 | 9.53 | 36.21 | 8.75 | 10.85 |
|  |  |  |  |  |  |
| **Confirmed Deaths** | 2.55 | 1.83 | 10.05 | 2.09 | 1.92 |
| 90% CI Lower | -2.20 | -3.36 | -11.41 | -1.79 | -2.10 |
| 90% CI Upper | 7.29 | 7.03 | 31.51 | 5.98 | 5.93 |

These are state-level, category-specific bivariable results regressing coefficients on the socioeconomic and epidemiological factors. Coefficient estimates for continuous variables represent the estimated effect of one standard deviation change in the predictor. A positive coefficient represents a smaller reduction in mobility. N=39. Robust standard errors are used.
