# Supplementary material for: Effectiveness of COVID-19 shelter-in-place orders varied by state
Source: PLoS One. 2020 Dec 31;15(12):e0245008. doi: 10.1371/journal.pone.0245008 (PMC7775080; doi:10.1371/journal.pone.0245008)
Supplement: S4 Table — This table reports z-statistics testing whether each state-pair in our sample has statistically different coefficients. Out of 741 tests, 429 (58%) are statistically different from one another. Green shading indicates a z-statistic greater than 1.96. (DOCX) [file pone.0245008.s007.docx]

**S4 Table:** Z-statistics for difference in state-level coefficients

|  | **AL** | **AK** | **AZ** | **CA** | **CO** | **DE** | **FL** | **GA** | **HI** | **ID** | **IL** | **IN** | **KS** | **LA** | **ME** | **MD** | **MA** | **MI** | **MN** | **MS** | **MO** | **MT** | **NV** | **NH** | **NJ** | **NM** | **NY** | **NC** | **OH** | **OR** | **PA** | **RI** | **SC** | **TN** | **VT** | **VA** | **WA** | **WV** |
| --- | --- | --- | --- | --- | --- | --- | --- | --- | --- | --- | --- | --- | --- | --- | --- | --- | --- | --- | --- | --- | --- | --- | --- | --- | --- | --- | --- | --- | --- | --- | --- | --- | --- | --- | --- | --- | --- | --- |
| **AL** | 0.0 | 1.3 | 2.4 | 3.0 | 1.0 | 0.7 | 0.1 | 4.5 | 0.4 | 0.4 | 1.9 | 3.1 | 4.2 | 3.3 | 0.5 | 3.2 | 2.3 | 0.8 | 3.3 | 5.6 | 0.3 | 2.6 | 3.7 | 3.2 | 0.7 | 2.2 | 6.7 | 2.2 | 0.4 | 1.7 | 5.9 | 2.0 | 6.3 | 11.3 | 2.6 | 1.0 | 1.1 | 0.2 |
| **AK** |  | 0.0 | 0.9 | 0.4 | 1.7 | 0.7 | 1.2 | 0.5 | 0.7 | 1.3 | 2.1 | 0.1 | 1.0 | 0.2 | 1.0 | 0.4 | 2.7 | 0.8 | 2.9 | 1.7 | 1.1 | 0.7 | 1.6 | 1.0 | 0.7 | 0.3 | 4.8 | 2.2 | 1.4 | 2.2 | 1.4 | 2.4 | 1.7 | 3.7 | 0.4 | 0.8 | 1.8 | 1.2 |
| **AZ** |  |  | 0.0 | 0.8 | 2.7 | 1.7 | 2.3 | 0.7 | 1.6 | 2.3 | 3.3 | 1.3 | 0.1 | 1.0 | 2.2 | 0.8 | 3.6 | 1.9 | 4.0 | 0.5 | 2.3 | 0.3 | 0.7 | 0.1 | 1.8 | 0.8 | 5.8 | 3.4 | 2.5 | 3.2 | 0.2 | 3.0 | 0.5 | 2.5 | 0.7 | 2.0 | 2.8 | 2.3 |
| **CA** |  |  |  | 0.0 | 2.9 | 1.4 | 2.5 | 0.2 | 1.3 | 2.3 | 4.4 | 0.9 | 1.1 | 0.4 | 2.5 | 0.0 | 3.7 | 1.9 | 5.4 | 2.2 | 2.5 | 0.5 | 1.8 | 0.9 | 1.7 | 0.1 | 8.2 | 4.6 | 3.0 | 3.6 | 1.8 | 2.8 | 2.3 | 5.9 | 0.1 | 2.1 | 3.0 | 2.7 |
| **CO** |  |  |  |  | 0.0 | 1.3 | 0.9 | 3.5 | 1.0 | 0.4 | 0.2 | 2.7 | 3.8 | 2.9 | 1.3 | 3.0 | 1.4 | 1.4 | 1.2 | 4.7 | 1.2 | 2.8 | 3.8 | 3.3 | 1.4 | 2.5 | 3.7 | 0.3 | 0.7 | 0.6 | 4.6 | 1.6 | 4.9 | 7.7 | 2.7 | 1.6 | 0.1 | 1.1 |
| **DE** |  |  |  |  |  | 0.0 | 0.6 | 1.7 | 0.1 | 0.8 | 1.8 | 0.9 | 2.2 | 1.2 | 0.4 | 1.4 | 2.4 | 0.1 | 2.8 | 3.1 | 0.5 | 1.6 | 2.6 | 2.0 | 0.1 | 1.2 | 5.2 | 1.9 | 0.9 | 1.9 | 2.9 | 2.2 | 3.2 | 5.9 | 1.3 | 0.1 | 1.3 | 0.5 |
| **FL** |  |  |  |  |  |  | 0.0 | 3.3 | 0.4 | 0.4 | 1.6 | 2.2 | 3.5 | 2.5 | 0.3 | 2.6 | 2.3 | 0.6 | 2.8 | 4.7 | 0.2 | 2.3 | 3.4 | 2.9 | 0.6 | 2.0 | 5.8 | 1.8 | 0.4 | 1.6 | 4.7 | 2.0 | 5.1 | 8.8 | 2.2 | 0.7 | 1.0 | 0.1 |
| **GA** |  |  |  |  |  |  |  | 0.0 | 1.6 | 2.8 | 6.3 | 1.8 | 1.1 | 0.8 | 3.7 | 0.3 | 4.1 | 2.6 | 7.1 | 2.5 | 3.6 | 0.4 | 1.8 | 0.8 | 2.2 | 0.3 | 10.3 | 6.5 | 4.1 | 4.2 | 2.2 | 2.9 | 2.8 | 7.9 | 0.1 | 3.1 | 3.5 | 4.0 |
| **HI** |  |  |  |  |  |  |  |  | 0.0 | 0.6 | 1.3 | 0.9 | 2.0 | 1.2 | 0.2 | 1.3 | 2.1 | 0.1 | 2.2 | 2.7 | 0.2 | 1.5 | 2.5 | 1.8 | 0.1 | 1.1 | 4.3 | 1.5 | 0.6 | 1.5 | 2.5 | 2.0 | 2.8 | 5.0 | 1.3 | 0.1 | 1.1 | 0.3 |
| **ID** |  |  |  |  |  |  |  |  |  | 0.0 | 0.7 | 2.0 | 3.1 | 2.3 | 0.7 | 2.4 | 1.7 | 0.9 | 1.7 | 4.0 | 0.6 | 2.3 | 3.3 | 2.8 | 0.8 | 2.0 | 4.1 | 0.9 | 0.1 | 1.0 | 3.8 | 1.8 | 4.2 | 6.8 | 2.2 | 1.0 | 0.5 | 0.5 |
| **IL** |  |  |  |  |  |  |  |  |  |  | 0.0 | 5.0 | 5.6 | 5.0 | 2.2 | 4.7 | 1.5 | 2.3 | 1.6 | 7.0 | 2.0 | 3.6 | 4.6 | 4.3 | 2.0 | 3.4 | 5.1 | 0.3 | 1.3 | 0.6 | 7.5 | 1.6 | 7.8 | 12.7 | 3.8 | 2.7 | 0.1 | 2.0 |
| **IN** |  |  |  |  |  |  |  |  |  |  |  | 0.0 | 2.3 | 0.7 | 2.3 | 1.0 | 3.5 | 1.4 | 6.1 | 3.8 | 2.3 | 1.2 | 2.5 | 1.7 | 1.2 | 0.6 | 9.4 | 5.3 | 2.9 | 3.4 | 3.8 | 2.6 | 4.3 | 9.8 | 0.9 | 1.7 | 2.7 | 2.6 |
| **KS** |  |  |  |  |  |  |  |  |  |  |  |  | 0.0 | 1.6 | 3.7 | 1.1 | 4.4 | 2.9 | 6.5 | 1.1 | 3.7 | 0.2 | 1.0 | 0.0 | 2.6 | 1.0 | 9.2 | 5.8 | 4.1 | 4.4 | 0.6 | 3.2 | 1.1 | 4.6 | 0.9 | 3.3 | 3.8 | 3.9 |
| **LA** |  |  |  |  |  |  |  |  |  |  |  |  |  | 0.0 | 2.6 | 0.4 | 3.7 | 1.8 | 6.0 | 2.9 | 2.7 | 0.8 | 2.1 | 1.3 | 1.6 | 0.2 | 9.1 | 5.2 | 3.1 | 3.6 | 2.7 | 2.7 | 3.3 | 7.9 | 0.4 | 2.1 | 3.0 | 2.9 |
| **ME** |  |  |  |  |  |  |  |  |  |  |  |  |  |  | 0.0 | 2.6 | 2.5 | 0.4 | 3.6 | 5.0 | 0.1 | 2.3 | 3.4 | 2.9 | 0.4 | 1.9 | 6.8 | 2.5 | 0.7 | 2.0 | 5.1 | 2.1 | 5.6 | 10.2 | 2.2 | 0.5 | 1.3 | 0.3 |
| **MD** |  |  |  |  |  |  |  |  |  |  |  |  |  |  |  | 0.0 | 3.8 | 1.9 | 5.7 | 2.3 | 2.7 | 0.6 | 1.8 | 0.9 | 1.7 | 0.1 | 8.5 | 4.9 | 3.1 | 3.7 | 1.9 | 2.8 | 2.5 | 6.3 | 0.1 | 2.2 | 3.0 | 2.9 |
| **MA** |  |  |  |  |  |  |  |  |  |  |  |  |  |  |  |  | 0.0 | 2.6 | 0.7 | 5.0 | 2.4 | 3.6 | 4.4 | 4.0 | 2.5 | 3.4 | 1.3 | 1.4 | 2.1 | 0.9 | 4.9 | 0.7 | 5.2 | 7.1 | 3.6 | 2.7 | 1.4 | 2.4 |
| **MI** |  |  |  |  |  |  |  |  |  |  |  |  |  |  |  |  |  | 0.0 | 3.5 | 4.1 | 0.5 | 1.9 | 3.0 | 2.4 | 0.0 | 1.5 | 6.4 | 2.5 | 1.0 | 2.1 | 4.0 | 2.2 | 4.4 | 8.2 | 1.7 | 0.0 | 1.5 | 0.6 |
| **MN** |  |  |  |  |  |  |  |  |  |  |  |  |  |  |  |  |  |  | 0.0 | 7.8 | 3.3 | 4.4 | 5.3 | 5.2 | 3.1 | 4.3 | 3.3 | 1.4 | 2.6 | 0.5 | 8.2 | 1.1 | 8.5 | 12.8 | 4.7 | 3.9 | 1.2 | 3.3 |
| **MS** |  |  |  |  |  |  |  |  |  |  |  |  |  |  |  |  |  |  |  | 0.0 | 5.0 | 1.0 | 0.3 | 0.8 | 3.7 | 1.9 | 10.4 | 7.2 | 5.3 | 5.3 | 0.6 | 3.5 | 0.0 | 3.5 | 1.8 | 4.6 | 4.7 | 5.3 |
| **MO** |  |  |  |  |  |  |  |  |  |  |  |  |  |  |  |  |  |  |  |  | 0.0 | 2.3 | 3.4 | 2.9 | 0.5 | 2.0 | 6.5 | 2.3 | 0.6 | 1.9 | 5.1 | 2.1 | 5.5 | 9.9 | 2.2 | 0.6 | 1.2 | 0.1 |
| **MT** |  |  |  |  |  |  |  |  |  |  |  |  |  |  |  |  |  |  |  |  |  | 0.0 | 1.1 | 0.2 | 1.8 | 0.5 | 6.5 | 3.7 | 2.7 | 3.3 | 0.7 | 2.9 | 1.0 | 3.4 | 0.4 | 2.0 | 2.8 | 2.4 |
| **NV** |  |  |  |  |  |  |  |  |  |  |  |  |  |  |  |  |  |  |  |  |  |  | 0.0 | 0.9 | 2.9 | 1.7 | 7.3 | 4.7 | 3.7 | 4.2 | 0.7 | 3.5 | 0.4 | 1.7 | 1.6 | 3.1 | 3.8 | 3.5 |
| **NH** |  |  |  |  |  |  |  |  |  |  |  |  |  |  |  |  |  |  |  |  |  |  |  | 0.0 | 2.2 | 0.9 | 7.5 | 4.5 | 3.3 | 3.8 | 0.4 | 3.1 | 0.8 | 3.5 | 0.8 | 2.6 | 3.3 | 3.1 |
| **NJ** |  |  |  |  |  |  |  |  |  |  |  |  |  |  |  |  |  |  |  |  |  |  |  |  | 0.0 | 1.4 | 5.9 | 2.2 | 0.9 | 2.0 | 3.5 | 2.2 | 3.9 | 7.1 | 1.6 | 0.0 | 1.4 | 0.6 |
| **NM** |  |  |  |  |  |  |  |  |  |  |  |  |  |  |  |  |  |  |  |  |  |  |  |  |  | 0.0 | 6.8 | 3.6 | 2.3 | 3.1 | 1.5 | 2.7 | 2.0 | 4.7 | 0.1 | 1.6 | 2.5 | 2.1 |
| **NY** |  |  |  |  |  |  |  |  |  |  |  |  |  |  |  |  |  |  |  |  |  |  |  |  |  |  | 0.0 | 4.8 | 5.8 | 3.0 | 11.1 | 0.1 | 11.3 | 15.3 | 7.3 | 7.1 | 3.6 | 6.6 |
| **NC** |  |  |  |  |  |  |  |  |  |  |  |  |  |  |  |  |  |  |  |  |  |  |  |  |  |  |  | 0.0 | 1.5 | 0.4 | 7.7 | 1.5 | 8.0 | 12.9 | 4.0 | 2.9 | 0.3 | 2.2 |
| **OH** |  |  |  |  |  |  |  |  |  |  |  |  |  |  |  |  |  |  |  |  |  |  |  |  |  |  |  |  | 0.0 | 1.4 | 5.4 | 1.9 | 5.8 | 10.0 | 2.7 | 1.2 | 0.8 | 0.5 |
| **OR** |  |  |  |  |  |  |  |  |  |  |  |  |  |  |  |  |  |  |  |  |  |  |  |  |  |  |  |  |  | 0.0 | 5.2 | 1.3 | 5.6 | 8.3 | 3.3 | 2.3 | 0.5 | 1.8 |
| **PA** |  |  |  |  |  |  |  |  |  |  |  |  |  |  |  |  |  |  |  |  |  |  |  |  |  |  |  |  |  |  | 0.0 | 3.4 | 0.6 | 4.9 | 1.5 | 4.6 | 4.6 | 5.5 |
| **RI** |  |  |  |  |  |  |  |  |  |  |  |  |  |  |  |  |  |  |  |  |  |  |  |  |  |  |  |  |  |  |  | 0.0 | 3.5 | 4.5 | 2.8 | 2.2 | 1.5 | 2.1 |
| **SC** |  |  |  |  |  |  |  |  |  |  |  |  |  |  |  |  |  |  |  |  |  |  |  |  |  |  |  |  |  |  |  |  | 0.0 | 4.0 | 1.9 | 5.1 | 4.9 | 5.9 |
| **TN** |  |  |  |  |  |  |  |  |  |  |  |  |  |  |  |  |  |  |  |  |  |  |  |  |  |  |  |  |  |  |  |  |  | 0.0 | 4.9 | 9.5 | 7.7 | 10.5 |
| **VT** |  |  |  |  |  |  |  |  |  |  |  |  |  |  |  |  |  |  |  |  |  |  |  |  |  |  |  |  |  |  |  |  |  |  | 0.0 | 1.8 | 2.8 | 2.4 |
| **VA** |  |  |  |  |  |  |  |  |  |  |  |  |  |  |  |  |  |  |  |  |  |  |  |  |  |  |  |  |  |  |  |  |  |  |  | 0.0 | 1.6 | 0.7 |
| **WA** |  |  |  |  |  |  |  |  |  |  |  |  |  |  |  |  |  |  |  |  |  |  |  |  |  |  |  |  |  |  |  |  |  |  |  |  | 0.0 | 1.1 |
| **WV** |  |  |  |  |  |  |  |  |  |  |  |  |  |  |  |  |  |  |  |  |  |  |  |  |  |  |  |  |  |  |  |  |  |  |  |  |  | 0.0 |

This table reports z-statistics testing whether each state-pair in our sample has statistically different coefficients. Out of 741 tests, 429 (58%) are statistically different from one another. Green shading indicates a z-statistic greater than 1.96.
