# Supplementary material for: Effectiveness of COVID-19 shelter-in-place orders varied by state
Source: PLoS One. 2020 Dec 31;15(12):e0245008. doi: 10.1371/journal.pone.0245008 (PMC7775080; doi:10.1371/journal.pone.0245008)
Supplement: Attachment — Submitted filename: CoverLetter_R1.pdf [file pone.0245008.s011.pdf]

December 12, 2020

Dear Dr. Meliker,

Thank you for again giving me the opportunity to serve as a referee for the *PLOS ONE*. Enclosed please find my referee report for “Effectiveness of COVID-19 Shelter-in-Place Orders Varied By States” (PONE-D-20-29575_R1).

The authors have addressed most of my comments and questions in the previous report. I do not have further comments.

As in my previous cover letter to you, I might not be in a position to judge if the contribution of this paper is sufficient for PLOS ONE. I hope my questions and comments are relevant and worth being addressed. Thank you.

Sincerely yours,

Ying-Ying Lee

Assistant Professor of Economics
University of California, Irvine
Irvine, CA 92697-5100, U.S.A.
yingying.lee@uci.edu
https://sites.google.com/site/yyleelilian/
