# Supplementary material for: Effectiveness of COVID-19 shelter-in-place orders varied by state
Source: PLoS One. 2020 Dec 31;15(12):e0245008. doi: 10.1371/journal.pone.0245008 (PMC7775080; doi:10.1371/journal.pone.0245008)
Supplement: Attachment — Submitted filename: report_R1.pdf [file pone.0245008.s012.pdf]

Report on PONE-D-20-29575_R1: “Effectiveness of COVID-19 Shelter-in-Place Orders Varied By State”

The authors have addressed most of my comments and questions in the previous report.

1. Regrading the first comment in my previous report: In Line 44 (now in Line 41), “SIP orders reduced mobility nationally by **12** percentage points...” I think it should be **12**, rather than **-12**? Doesn’t ”reduce...by -12” mean “increase...by 12”?

2. For the first robustness check, running the RD using the covariates as the dependent variable is called “*validity check*” in the literature, to my knowledge. This falsification test is for the identification assumptions to access whether the RD design is valid. This is different from checking the robustness of the results against various bandwidths.
