# Supplementary material for: Effectiveness of COVID-19 shelter-in-place orders varied by state
Source: PLoS One. 2020 Dec 31;15(12):e0245008. doi: 10.1371/journal.pone.0245008 (PMC7775080; doi:10.1371/journal.pone.0245008)
Supplement: Attachment — Submitted filename: PLOS review Oct 2 2020.docx [file pone.0245008.s009.docx]

I find the manuscript relevant, clear, and technically sound, for the most part. I would endorse its publication if the authors successfully address the points raised below.

The paper has three major findings: 1) the mean effect of shelter-in-place orders 2) the variance in those effects by state 3) a list of mediating variables.

The biggest threat, as I see it, to the analysis’s first two findings, with implications for the third, is that other time-varying COVID-relevant policies were simultaneously taking place, potentially confounding the analysis.

These include: school closures, closure/restrictions on public transportation, restrictions on gatherings, and the closures of non-essential businesses, to name just three types of policies that varied by state on a day-to-day basis during the time period of analysis. Each of these could affect mobility data.

At the very least, the authors need to devote some space to summarizing analysis and discussion of these policies and why they don’t need to be explicitly modeled alongside SIP orders. My sense is that SIP orders left non-essentially businesses open in some states (particularly Democratic Party-oriented states) but not others.

So, one thing the authors need to consider is that not all SIP orders took the same form, and that might explain some of the variation in efficacy on mobility.

There is one more serious threat to accurate identification: the disease burden. The Northeast (especially New York City) was hit harder and earlier than much of the rest of the country. I don’t see anything in the authors’ discussion or code that indicates that the disease burden (ideally measured as deaths per capita) was considered as a control variable alongside the SIP order, but surely, an explosion of deaths and infections in a local area or state would be a relevant consideration as to whether or not people feel comfortable venturing out—and whether or not a state governor or legislative body issues a SIP order in the first place.

Along these lines, there is a serious threat to endogeneity. The SIP orders were not issued randomly. Governors decided to issue them or not based on relevant political party alignment and the disease burden in their jurisdiction. I believe this issue needs to at least addressed under “limitations” and, potentially, the methods section, even if there is no analytic solution, other than robustness checks that include the disease burden in the main estimations.

Otherwise, I think the article is well structured, and I think the discussion and analysis of factors that distinguish effect sizes across states is valuable and consistent with other evidence on variation in compliance: <https://papers.ssrn.com/sol3/papers.cfm?abstract_id=3638373>
