# Supplementary material for: Effectiveness of COVID-19 shelter-in-place orders varied by state
Source: PLoS One. 2020 Dec 31;15(12):e0245008. doi: 10.1371/journal.pone.0245008 (PMC7775080; doi:10.1371/journal.pone.0245008)
Supplement: Attachment — Submitted filename: plos_covid_response_letter_final.docx [file pone.0245008.s010.docx]

Comments from editor

**1. Please ensure that your manuscript meets PLOS ONE's style requirements, including those for file naming. The PLOS ONE style templates can be found at**

**https://journals.plos.org/plosone/s/file?id=wjVg/PLOSOne_formatting_sample_main_body.pdf and**

**https://journals.plos.org/plosone/s/file?id=ba62/PLOSOne_formatting_sample_title_authors_affiliations.pdf**

We have updated all formatting to be consistent with these requirements.

**2. In the Methods section of the manuscript, please address the following:**

**-	Please provide a justification for why Donald J Trump was selected as the political proxy.**

We have added the following in the methods section:

“We believe this is an appropriate proxy of partisan political affiliation, as it captures not only political alignment but also potential agreement with Donald J. Trump’s negative expressed views on lockdowns and SIP orders (for example, the President allegedly considered “reopening” as early as March).”

**-	Please provide a citation for the AHRF database, the date ranges over which you conducted the search, which variables were studies. And ensure that you have provided sufficient details that others could replicate the analyses**

We have added the reference to the AHRF and have added the following text:

“We extracted the following county-level measures from the AHRF: population estimate (F11984-18); per-capita income (F09781-17); poverty rate (F13321-17); Black population (F13910-17); and total area in square miles (F13874-10). We created a population-density measure by dividing population by total area in square miles, and a share of Black residents by dividing Black population by total population.”

**3. Please provide additional details regarding participant consent. In the ethics statement in the Methods and online submission information, please ensure that you have specified (1) whether consent was informed and (2) what type you obtained (for instance, written or verbal, and if verbal, how it was documented and witnessed). If your study included minors, state whether you obtained consent from parents or guardians. If the need for consent was waived by the ethics committee, please include this information.**

**If you are reporting a retrospective study of medical records or archived samples, please ensure that you have discussed whether all data were fully anonymized before you accessed them and/or whether the IRB or ethics committee waived the requirement for informed consent. If patients provided informed written consent to have data from their medical records used in research, please include this information.**

**Once you have amended this/these statement(s) in the Methods section of the manuscript, please add the same text to the “Ethics Statement” field of the submission form (via “Edit Submission”).**

**For additional information about PLOS ONE ethical requirements for human subjects research, please refer to http://journals.plos.org/plosone/s/submission-guidelines#loc-human-subjects-research**

We have added the following ethics statement:

“Our study involved secondary analysis of non-identifiable, aggregate data and therefore is not human subjects research. No IRB approval was sought.”

**4. We note that you have included the phrase “data not shown” in your manuscript. Unfortunately, this does not meet our data sharing requirements. PLOS does not permit references to inaccessible data. We require that authors provide all relevant data within the paper, Supporting Information files, or in an acceptable, public repository. Please add a citation to support this phrase or upload the data that corresponds with these findings to a stable repository (such as Figshare or Dryad) and provide and URLs, DOIs, or accession numbers that may be used to access these data. Or, if the data are not a core part of the research being presented in your study, we ask that you remove the phrase that refers to these data.**

We have removed this and have added the numerical results in the text.:

“RD models weighted by county population produced similar estimates (-9.9, 95% CI:-13.2 to -6.7).”

**5. Please include captions for your Supporting Information files at the end of your manuscript, and update any in-text citations to match accordingly. Please see our Supporting Information guidelines for more information:** [**http://journals.plos.org/plosone/s/supporting-information**](http://journals.plos.org/plosone/s/supporting-information)**.**

We have added these captions, as well as additional supporting information files. We have also removed figures from the main manuscript and uploaded them separately.

Comments from Reviewer 1

**I find the manuscript relevant, clear, and technically sound, for the most part. I would endorse its publication if the authors successfully address the points raised below.**

**The paper has three major findings: 1) the mean effect of shelter-in-place orders 2) the variance in those effects by state 3) a list of mediating variables.**

**The biggest threat, as I see it, to the analysis’s first two findings, with implications for the third, is that other time-varying COVID-relevant policies were simultaneously taking place, potentially confounding the analysis.**

**These include: school closures, closure/restrictions on public transportation, restrictions on gatherings, and the closures of non-essential businesses, to name just three types of policies that varied by state on a day-to-day basis during the time period of analysis. Each of these could affect mobility data.**

**At the very least, the authors need to devote some space to summarizing analysis and discussion of these policies and why they don’t need to be explicitly modeled alongside SIP orders. My sense is that SIP orders left non-essentially businesses open in some states (particularly Democratic Party-oriented states) but not others.**

**So, one thing the authors need to consider is that not all SIP orders took the same form, and that might explain some of the variation in efficacy on mobility.**

We thank the reviewer for taking the time to review our manuscript and suggesting ways to improve upon our work. We agree that each state has a unique policy environment which may moderate/mediate the effectiveness of state SIP orders. While a review of these myriad policies is outside the scope of this text, we have clarified in the text that: (a) SIP orders may have occurred alongside other restrictions implemented at the same time and our results should be interpreted as reflecting the aggregate impact of all policies implemented simultaneously with SIPs (b) implementation of other policies at the same time (e.g. closures of transportation, non-essential businesses, etc.) could have encouraged and enabled greater compliance with the SIPs and could be one factor explaining differences across states. In our revision, we ran the following analysis: using the BU database of policy responses, we obtained information on whether there were school closures, daycare closures, nursing home restrictions, business closures, restaurant closures, and gym closures. We then ran several regressions of our estimates of SIP effectiveness on indicators for these policies. Only school and non-essential business closures were associated with variation in the RD estimate, both being associated with more effective SIP orders. Notably, only one state did not enact a school closure in our time period (California). We have added the following paragraph in the results section:

“We found some evidence that additional policies modified the effectiveness of SIP orders. School closures (-5.7%, 90% CI: -8.4 to -2.9) and non-essential business closures (-4.9%, 90% CI: -9.6 to -0.1) appear to be associated with more effective SIP orders. No other policies were significantly associated with variation in effectiveness of state-level SIP orders. (Table 2)”

We have added the following to the limitations section:

“Third, other policies (such as transportation or business closures) were implemented either in tandem with or prior to SIP order enactment. These policies likely had their own effects, and may have affected compliance with SIP orders, which may explain some of the variation between states.”

**There is one more serious threat to accurate identification: the disease burden. The Northeast (especially New York City) was hit harder and earlier than much of the rest of the country. I don’t see anything in the authors’ discussion or code that indicates that the disease burden (ideally measured as deaths per capita) was considered as a control variable alongside the SIP order, but surely, an explosion of deaths and infections in a local area or state would be a relevant consideration as to whether or not people feel comfortable venturing out—and whether or not a state governor or legislative body issues a SIP order in the first place.**

The reviewer highlights an important point. Unlike standard linear or logistic regression approaches, effect estimates regression discontinuity designs are not biased by covariates unless there are discontinuities in average covariate values at the threshold (Athey & Imbens, Journal of Economic Perspectives, 2017). We thus tested for these discontinuities in the revised manuscript:

Even without such a discontinuity, disease burden may be an important moderating factor for SIP effectiveness. In the revised version, we

**Along these lines, there is a serious threat to endogeneity. The SIP orders were not issued randomly. Governors decided to issue them or not based on relevant political party alignment and the disease burden in their jurisdiction. I believe this issue needs to at least addressed under “limitations” and, potentially, the methods section, even if there is no analytic solution, other than robustness checks that include the disease burden in the main estimations.**

This is an important point. We agree with the reviewer that enactment of SIP orders is likely endogenous to disease conditions and political considerations. Although this is a problem in most analyses of SIPs, the regression discontinuity approach offers a more compelling and better identified way to get at policy impact (see response to previous comment). We identify solely off of the discontinuous change that occurs on the date of SIP implementation. Therefore, while pre-SIP conditions could spur the implementation of a SIP, the change in mobility immediately after a SIP is put into place would be unlikely to occur in the absence of the SIP. We have clarified that this is a strength of our analysis in the discussion section as follows:

“Notably, a strength of our approach is that while policy enactment might be endogenous to pre-policy conditions, our estimates are identified entirely from the discontinuous change that occurs post-enactment.

We have added the following to our results section:

“To test for whether disease burden is a confounder, we examined whether it has a discontinuity with the running variable. Our results (0.48, 95% CI:-0.04 to 0.99) indicate that while it is plausible that SIP enactment is endogenous to disease burden, it is nonetheless not a confounder for our analysis.”

**Otherwise, I think the article is well structured, and I think the discussion and analysis of factors that distinguish effect sizes across states is valuable and consistent with other evidence on variation in compliance:** [**https://papers.ssrn.com/sol3/papers.cfm?abstract_id=3638373**](https://papers.ssrn.com/sol3/papers.cfm?abstract_id=3638373)

We thank the reviewer for these helpful comments and have added the reference in our revision.

Comments from Reviewer 2

**Other drivers of heterogeneity. There are several aspects of the analysis that may cause heterogeneous estimates even if the effect of the policies are the same.
- The RD bandwidth differs across states. Moreover, the "private" or voluntary distancing behaviors may differ across these bandwidths. It would be good to (a) examine robustness to a fixed bandwidth for the RD, and (b) examine robustness to using an event-study specification as in Allcott et al. (2020; "Economic and Health Impacts of Social Distancing Policies during the Coronavirus Pandemic"). E.g., What is the correlation between the current state-level estimates and the state-level estimates from (a) and (b)?**

We agree that the use of different bandwidths across states may add variability to our estimates. We have re-run each state model using the fixed bandwidth of 2.9 that was used for the overall model. The results are presented below, and are highly correlated (r=0.77). We believe that allowing for variable data-driven bandwidths is more appropriate as this approach allows for more flexibility in estimating state-specific levels of mobility immediately before and immediately after SIP implementation. Prior work has illustrated that data-driven bandwidth selection tends to outperform alternative approaches. (Calonico, Cattaneo, and Titunik 2014) That is, use of variable bandwidths is consistent with the notion of identification at the threshold in an RDD design.

We have added the following to the statistical analysis section:

“Second, while data-driven bandwidths tend to be superior to alternative bandwidth selection procedures, (21) it is possible for variation in state-level results to arise due to variation in bandwidths. To account for this, we re-ran our RD model for each state with a fixed bandwidth taken from our primary model. We then examined whether the estimated coefficients are correlated with the estimated from our primary state-level RD specifications.”

In addition, we have added the figure below to the appendix, and have added the following to our results section:

“While estimates from state-level models with a fixed bandwidth differed from estimates that allowed the bandwidth to vary by state, the estimated coefficients were strongly correlated (r=0.77)”

We also thank the reviewer for pointing us to the recent work by Allcott et al. We do note that this is a fundamentally different identification strategy and requires different assumptions than our RD approach. We conducted two analyses:

First, we re-ran our RD specification with a uniform kernel and ran an event study specification without any fixed effects to estimate both models in as similar a manner as possible. In the RD approach the coefficient estimate is -11.1 (SE:0.38) and in the event study approach the coefficient estimate is equal (-11.1, SE: 0.38).

The event study graph is presented below.

Next, we replicated the event-study specification with the same restrictions (up to 21 leads/lags) and date and county fixed effects as used by Allcott et al. Here, the coefficient is smaller (-7.9, SE: 0.24) than our primary RD estimate (-12.03, SE: 0.84) but is similar in magnitude. We’ve presented the overall result below and have added it to the appendix.

We have also added the following to our results section:

“Replicating prior work that uses an event study design (15) we find that the estimate for one day after SIP implementation (-7.9, 95% CI: -8.5 to -7.5) is similar to our primary RD result. We note that whereas the event study compares means on either side of the threshold, the RD approach leverages data points away from the threshold in order to obtain more precise estimates of state-specific effects.”

**- The authors take the mean across categories in the Google Mobility Reports. Different states may have different shares of categories. It would be good to also examine each category individually as robustness to make sure the results agree. Again, what is the correlation between the state-level estimates using the mean index and the state-level estimates using each individual category?**

While we agree that in principle, separating out mobility for each category would be ideal, we aggregated to a mean in order to deal with missing data for many counties. For instance, only 44,000 county-day observations (out of 286,000) have non-missing data for visits to parks. Nonetheless, we examined each category individually and found that in general, the estimates across the categories are positively correlated with the exception of visits to the workplace. We hypothesize that this is because many essential workers continued to go to work.

In addition, we investigated whether variation in state-level estimates for these categories is associated with the various socioeconomic and epidemiological factors we identified for our primary analysis.

We have added the following to our statistical analysis section:

“Lastly, to understand how different categories of mobility (e.g., workplace, transit use etc.) responded to SIP orders, we re-ran our primary state-level RD specification with each mobility category as the outcome. We investigated variation in these coefficients across states, and investigated their association with the epidemiological and socioeconomic factors discussed earlier.”

We have added the following to our results section:

“We re-ran our primary RD specification using changes in mobility across each non-residential category separately and investigated the correlation of these measures. In general, all measures moved in the same direction as our index measure, and were generally strongly correlated with the index measure. The one exception was mobility at the workplace, which we hypothesize might be because essential workers continued to show up at work.”

As well as the following:

“As with our primary analysis, we find no relationship between cases or deaths for category-specific analyses. Consistent with the results for our index measure of mobility, Donald J. Trump vote share was associated with smaller reductions in mobility for parks (-20.8, 90% CI: -35.8 to -5.9) and transit (-3.45, 95% CI: -6.4 to -0.6). Our findings were similar for per capita income and poverty rate for certain categories as well. A unique finding from this analysis is that early SIP implementation appears to be associated with a large decline in mobility in the workplace category (7.54, 90% CI: 0.29 to 14.79).”

We have added the following paragraph to our discussion section:

“However, in examining category-specific mobility changes, we do find that earlier SIP implementation appears to be associated with larger declines in visits to workplaces. While we cannot identify the precise mechanism for this, it may be that some private responses may have been counter to the public response (e.g., employers attempting to stay open).”

And we have added the tables below to the appendix:

**Mean State-level RD Estimate by Category**

| **Category** | **Mean Estimate** | **Mean 95% Lower CI** | **Mean 95% Upper CI** |
| --- | --- | --- | --- |
| Overall (N=17,682) | -11.8 | -17.5 | -6.1 |
| Recreation (N=16,073) | -8.8 | -14.6 | -3.1 |
| Grocery Stores (N=12,639) | -13.7 | -20.2 | -7.2 |
| Parks (N=5,543) | -18.5 | -56.3 | 19.9 |
| Transit (N=11,211) | -4.2 | -16.9 | 8.5 |
| Workplace (N=13,093) | -8.1 | -12.5 | -3.8 |

**Correlation Matrix of Estimates by Category**

|  | Overall | Recreation | Grocery Stores | Parks | Transit | Workplace |
| --- | --- | --- | --- | --- | --- | --- |
| Overall | 1 |  |  |  |  |  |
| Recreation | 0.52 | 1 |  |  |  |  |
| Grocery Stores | 0.63 | 0.77 | 1 |  |  |  |
| Parks | 0.71 | 0.44 | 0.35 | 1 |  |  |
| Transit | 0.46 | 0.75 | 0.49 | 0.39 | 1 |  |
| Workplace | -0.01 | -0.59 | -0.32 | -0.10 | -0.45 | 1 |

**Mobility category-specific bivariable results**

|  | **Recreation** | **Grocery Stores** | **Parks** | **Transit** | **Workplace** |
| --- | --- | --- | --- | --- | --- |
| **Trump Vote Share** | 2.45 | 2.22 | 20.82 | 3.45 | -0.07 |
| 90% CI Lower | -0.75 | -2.48 | 5.87 | 0.55 | -2.56 |
| 90% CI Upper | 5.66 | 6.91 | 35.78 | 6.36 | 2.43 |
|  |  |  |  |  |  |
| **Pop Density** | -0.08 | -5.57 | -13.46 | -0.51 | 0.34 |
| 90% CI Lower | -0.80 | -6.39 | -16.36 | -1.11 | -0.12 |
| 90% CI Upper | 0.64 | -4.75 | -10.56 | 0.10 | 0.79 |
|  |  |  |  |  |  |
| **Early SIP** | 4.61 | 3.01 | -33.62 | 3.13 | -7.54 |
| 90% CI Lower | -7.01 | -9.77 | -71.45 | -9.06 | -14.79 |
| 90% CI Upper | 16.22 | 15.80 | 4.20 | 15.32 | -0.29 |
|  |  |  |  |  |  |
| **Per Capita Income** | -2.23 | -4.65 | -17.37 | -3.08 | 1.24 |
| 90% CI Lower | -5.27 | -8.78 | -25.57 | -5.78 | -1.33 |
| 90% CI Upper | 0.81 | -0.53 | -9.18 | -0.38 | 3.80 |
|  |  |  |  |  |  |
| **Poverty Rate** | 4.28 | 3.23 | 8.27 | 4.06 | -1.51 |
| 90% CI Lower | 1.05 | -1.11 | -3.19 | 1.24 | -4.17 |
| 90% CI Upper | 7.51 | 7.56 | 19.73 | 6.87 | 1.14 |
|  |  |  |  |  |  |
| **% Black** | 2.22 | 0.91 | 0.66 | 3.33 | -0.29 |
| 90% CI Lower | -1.87 | -4.20 | -9.84 | -0.55 | -3.11 |
| 90% CI Upper | 6.32 | 6.03 | 11.17 | 7.21 | 2.53 |
|  |  |  |  |  |  |
| **Confirmed Cases** | 2.21 | -1.11 | -2.37 | 2.52 | 4.15 |
| 90% CI Lower | -5.15 | -11.74 | -40.96 | -3.70 | -2.56 |
| 90% CI Upper | 9.58 | 9.53 | 36.21 | 8.75 | 10.85 |
|  |  |  |  |  |  |
| **Confirmed Deaths** | 2.55 | 1.83 | 10.05 | 2.09 | 1.92 |
| 90% CI Lower | -2.20 | -3.36 | -11.41 | -1.79 | -2.10 |
| 90% CI Upper | 7.29 | 7.03 | 31.51 | 5.98 | 5.93 |

These are state-level, category-specific bivariable results regressing coefficients on the socioeconomic and epidemiological factors. Coefficient estimates for continuous variables represent the estimated effect of one standard deviation change in the predictor. A positive coefficient represents a smaller reduction in mobility. N=39. Robust standard errors are presented.

**- The authors do not account for county-level SIPs. Different states may have different shares of county SIPs already---driving observed heterogeneity. See Allcott et al. (2020; "Economic and Health Impacts of Social Distancing Policies during the Coronavirus Pandemic") for treatment of county level SIPs.**

While we agree with the reviewer that county-level SIP orders may have had some county-specific effects, this would not bias our results because once a state SIP is enacted, all counties become “treated.” County-level SIP orders certainly may have played a role in moderating the effectiveness of state-level SIP (likely reducing their effectiveness). However, because of the relatively small number of county-level SIP orders (a little over 300 in Allcott 2020) and the challenge in incorporating them correctly into an RD framework, we don’t believe that additional analyses is warranted.

We have added the following to our limitations section:

“Fourth, while some other work has relied on county-level SIP orders as well, our analysis has not accounted for these changes. However, because state-level SIP orders affect all counties within the state, county-level SIP orders are unlikely to bias our results. Rather, county-level SIP orders may act as a moderator possibly reducing the effectiveness of state-level SIP orders.”

**Correlates of estimated treatment effects.**
**- Small sample size is not a good reason to change the standard of statistical significance. The authors should just report the coefficient estimates along with the standard errors. The reader can then easily perform any required statistical tests.**

While we recognize that the appropriate reporting of confidence intervals and determination of statistical significance is a contentious issue, we disagree that small sample size is not an adequate reason to rely on a narrower confidence interval. Even R.A. Fisher (1925) suggested alpha levels of 0.10 may be appropriate for small samples. First, by using a narrower confidence interval, we reduce the margin of error of the conditional mean. Second, if we did use the full sample size of counties and weighted by population, it is likely that most of our bivariable regressions would (spuriously) produce statistically significant results.

**- It would be useful to see multivariate analysis of all covariates in addition to the bivariate analysis that is already conducted.**

Unfortunately, due sample size limitations (N=39), we do not have enough observations for the regression to run successfully with all covariates included on the right-hand side of the equation.

**The authors should really have standard RD plots for each state estimate in the appendix so the reader can visualize the extent to which they think the RD assumptions are valid.**

We agree and have added a matrix of RD plots to the appendix.

**Literature:
- Painter and Qiu (2020; "Political beliefs affect compliance with COVID-19 Social Distancing Orders") should be cited because it is most relevant to examining heterogenous effects in response to SIPs by partisanship.
- Please also go through** [**https://scholar.google.com/scholar?cites=939500896822965987**](https://scholar.google.com/scholar?cites=939500896822965987) **to make sure all relevant literature is being cited for heterogenous effects of SIPs (e.g., Wright et al.).
- The findings in this manuscript should then be compared to what others have found.**

We have added these citations and have added to our discussion some framing in context of what others have found.

“In addition, while not directly comparable, our analysis is consistent with recent work finding that income is associated with the ability to comply with social distancing orders. (12) Our results are also similar in magnitude to other work using event-study designs (22), and we come to similar conclusions that a large component of the overall mobility reduction occurred due to a private rather than policy-driven response.”

Comments from Reviewer 3

**1. In Line 44, "SIP orders reduced mobility nationally by 12 percentage points."**

There may have been text cut off; we are unsure of the reviewer’s specific concern but may incorporate any additional feedback in the next iteration.

**2. The observations are Android cellphone users who have turned on location tracking. Are these observations representative of the population? Could the authors provide some summary statistics of demographics of such a sub-population?**

Unfortunately, demographics of this subpopulation are not accessible to us. We have noted in the limitations section that the demographics of individuals represented by Google mobility data may not be representative of the overall population. In addition, we have referenced a [report](http://www.fluentco.com/wp-content/uploads/2016/01/Fluent2_DevicesandDemographics_2016.pdf) documenting some important demographic differences between Android and iPhone users.

We have added the following to the limitations section:

“Second, it is unclear whether the Android users who contribute data to Google Community Mobility Reports are representative of individuals living in that county. Unfortunately, this information is unavailable. Indeed, there are observed demographic differences between Android and iPhone users across age groups and gender.(24) To the extent they are different, our results may not generalize to other individuals.”

**3. In Line 120, it is unclear how the index measure is constructed. The index measure is the mean of what?**

We have clarified in line 115-118 that this is the mean of the percent change in mobility for all non-residential categories:

“We then computed an index measure by taking the mean of percent change for all non-residential categories which included: retail and recreation, groceries and pharmacies, parks, transit stations, and workplaces.”

**4. In Line 129, the observations on the day of implementation are dropped. I wonder how the estimation results could change without dropping those observations. The SIP orders should be expected, so I am not sure if the washout period should be taken into account. The authors may give more justication or reference.**

While we agree with the reviewer that in some cases, policy changes are anticipated, we do not believe this is the case with SIP orders. California, for instance, announced its SIP order on the same day that it was to be enacted (<https://www.kqed.org/science/1959566/california-gov-gavin-newsom-orders-state-to-shelter-in-place>). Massachusetts was in a similar situation (<https://www.mass.gov/news/governor-charlie-baker-orders-all-non-essential-businesses-to-cease-in-person-operation>). In these cases, a washout period accounts for potential short-term increase in mobility as people rush the stores (<https://fortune.com/2020/04/20/coronavirus-retail-industry-ecommerce-online-shopping-brick-and-mortar-covid-19/>), and also allows for a short time for people to respond to the order appropriately. We have added the following in the Study Variables section:

“In addition, when assessing other policies, there may be expectation of enactment. SIP orders, however, appear to have been announced with little to no delay between announcement and enactment.(9) Thus the announcement of these orders were associated with spikes in mobility as residents rushed to stock up on essential goods. (10) To account for this, and to capture actual response to the SIP order we allowed for a one-day washout period. We thus dropped observations on the day of implementation.”

**5. In Figure 2, the label of the horizontal axis is \Days from SIP Implementation." Shouldn't it be \percentage change in mobility"? I don't see how the relative date of SIP implementation reflects in the figure. Is California the last to implement SIP, and is South Carolina the first?**

We appreciate the opportunity to clarify. The reviewer is correct that the x-axis should say “Percentage Change in Mobility,” and we have modified it accordingly. The reviewer is also correct that the figure does not represent the relative date of implementation, and that this caption was meant to refer to Figure 1. We have modified both figures accordingly.

Comments from Reviewer 4

**Major comments:

* An important factor that may explain the findings are differences in how the SIP and related measures were implemented in each state. For example, some states had more enforcement, more stringent measures such as restrictions on religious and other public events, maximum number of people allowed in social gatherings, closing of different types of businesses (indoor and outdoor dining, bars, gyms, stores), etc. Policies aiming at restricting mobility also differed widely at the county and city level in each state. Hence, part of the difference in effects may be due to differences in how the SIP and accompanying policies were implemented across states. The estimated differences across states may therefore represent responses to different policies (or bundles of policies), not differential responses to a homogeneous policy implemented uniformly across states.**

We agree with the reviewer that details of policy implementation may have contributed to differences in effectiveness across states. This is related to a comment that Reviewer 1 made, noting that other policies were also enacted during this time. In an ideal scenario, we would have multiple components of the policy itself coded and be able to control for it in analysis. Because this is impractical, we investigated whether the state-level coefficients we estimated vary with indicators of other policies (school closures, daycare closures, nursing home restrictions, business closures, restaurant closures, and gym closures). Across these regressions, at the 90% confidence interval, only school and business closures were associated with variation in the RD estimate, both being associated with more effective SIP orders.

We have added the following to the statistical analysis section:

“Because SIP orders were implemented alongside complementary policies in some states, we also assessed whether SIP impact differed by whether the state had implemented a range of other measures at the time of the SIP order.”

We have added this as a robustness check in the manuscript:

“We found some evidence that additional policies modified the effectiveness of SIP orders. School closures (-5.7%, 90% CI: -8.4 to -2.9) and non-essential business closures (-4.9%, 90% CI: -9.6 to -0.1) appear to be associated with more effective SIP orders. No other policies were significantly associated with variation in effectiveness of state-level SIP orders. (Table 2)”

We have added the following to the discussion section:

“Less surprisingly, we found that certain policies – school closures and non-essential business closures – were complementary to SIP orders. It may be that these policies reduce burdens that would otherwise limit the ability of individuals to adhere with SIP orders.”

We have added the following to the limitations section:

“Third, other policies (such as transportation or business closures) were implemented either in tandem with or prior to SIP order enactment. These policies likely had their own effects, and may have affected compliance with SIP orders, which may explain some of the variation between states.”

*** The differences between the estimates of mobility changes across states do not seem statistically significant in most cases (Figure 2). Hence much of this estimated variation may be due to sampling variability, not necessarily to real heterogeneity in the effect of the SIP orders.**

While the reviewer is correct that there are a number of states where the effect of SIP orders was non-statistically significant, this is the case in one-quarter of states in this sample (10 out of 39). In addition, the results in our overall model is also driven by within-state variation..

*** The regressions shown in Table 1 use only 39 observations, which seems very low. Lack of significance in the results for epidemiological factors may be due to lack of statistical power and not a true lack of effect. The statistical analysis does not convincingly justify the conclusions on page 12 (lines 265-273).**

While we agree with the reviewer that small sample size may present an issue (and this is why we report 90% confidence intervals), we believe that our conclusion that post-SIP mobility changes were not simply a continuation of an existing trend are still valid. Indeed, we did find that certain moderating factors were significant, and those that weren’t significant had an estimated effect size closer to zero. This is broadly consistent with the visual inspection of the RD plot in Figure 1 and with our bivariable analysis.

**Minor comments:

* The data only include Android cellphone users. The authors should discuss whether they think this subgroup is representative of each state's population.**

We agree that this is of possible concern. We have added a reference to an analysis of demographic differences between iPhone and Android users. We have also added the following discussion in the limitations section:

“Second, it is unclear whether the Android users who contribute data to Google Community Mobility Reports are representative of individuals living in that county. Unfortunately, this information is unavailable. Indeed, there are observed demographic differences between Android and iPhone users across age groups and gender.(24) To the extent they are different, our results may not generalize to other individuals.”

*** I was confused by the second paragraph on page 8 and the note under Table 1 stating that standard errors are clustered at the state level. My understanding is that the data for these regressions is already at the state level, which means each cluster has a single observation (so standard errors are heteroskedasticity-robust but do not effectively allow for clustering in any useful way).**

The reviewer is correct, and we have corrected this typo.

*** It would be interesting to analyze changes in mobility after the SIP is lifted to see whether mobility reverts back to its previous levels or remains lower.**

We agree with the reviewer that this would be an interesting line of inquiry! Unfortunately, we leave this question to future researchers as an answer would require a substantial expansion of our existing analysis and is beyond the scope of our manuscript.
