# Supplementary material for: Effectiveness of COVID-19 shelter-in-place orders varied by state
Source: PLoS One. 2020 Dec 31;15(12):e0245008. doi: 10.1371/journal.pone.0245008 (PMC7775080; doi:10.1371/journal.pone.0245008)
Supplement: Attachment — Submitted filename: plos_covid_response_letter_final.docx [file pone.0245008.s013.docx]

Comments from Reviewer 2

**1. I won't argue with the authors about the appropriate significance level and whether it should vary with sample size. I do, however, think a simple solution to people having different views on this would be to just add the standard errors to Tables 1 and 2 in addition to the 90% CI.**

We agree with the reviewer that the appropriate significance level may be in the eye of the beholder. As suggested, we have added the standard errors to Tables 1 and 2.

**2. The point about county SIPs not biasing results depends on what the authors are trying to estimate. If the authors are trying to estimate the treatment effect of an entire state adopting a SIP relative to an entire state (including all counties) having no SIP, then pre-existing county SIPs are going to bias those estimates. I think the authors should drop claims regarding bias, and just note that pre-existing county SIPs may attenuate estimates.**

The reviewer’s point is well-taken. We have added the following to the limitations section:

“Any pre-existing county-level SIP orders may attenuate our effect estimates. Thus, our results may be a conservative estimate.”

Comments from Reviewer 3

**1. In Line 44 (now in Line 41), "SIP orders reduced mobility nationally by 12 percentage points..." I think it should be 12, rather than -12? Doesn't "reduce...by -12" mean \increase...by 12"?**

We thank the reviewer for catching this typo!

**2. For the first robustness check, running the RD using the covariates as the dependent variable is called "validity check" in the literature, to my knowledge. This falsification test is for the identification assumptions to access whether the RD design is valid. This is different from checking the robustness of the results against various bandwidths.**

We agree with the reviewer. We present both sets of analyses in our results and clarify their meaning:

In the results section we have written:

“While estimates from state-level models with a fixed bandwidth differed from estimates that allowed the bandwidth to vary by state, the estimated coefficients were strongly correlated (r=0.77)(Figure S2).”

As well as:

“To test whether disease burden is a confounder in our RD models, we examined whether it has a discontinuity with the running variable. Our results (0.48, 95% CI:-0.04 to 0.99) indicate that while it is plausible that SIP enactment is endogenous to disease burden, it is nonetheless not a confounder for our analysis.”

Comments from Reviewer 4

**The analysis of effect heterogeneity across states is still unconvincing. On the one hand, the authors estimate a large number of effects for different states, but do not provide any statistical test to show that the effects are significantly different between states. Here I do not mean testing whether the effects are significantly different from zero, which can easily be seen from the confidence intervals in Figure 2, but whether the effects are significantly different from each other, which is what the authors argue in their paper. In fact, most of the estimates in Figure 2 seem rather close to each other and their confidence intervals overlap in a large number of cases, which suggests that indeed many of these estimates may actually be capturing the same or very similar effects (perhaps with some exceptions such as Tennessee). Given that effect heterogeneity is the main point of this paper, the analysis of this issue seems overly superficial.**

The reviewer makes an important point; we had relied on the ‘eye test’ showing that many state confidence intervals do not overlap, and had not formally tested whether state-level estimates are statistically different from one another. For this revision, we conducted Z-tests comparing regression coefficients for every state pairing (see Clogg, C. C., Petkova, E., & Haritou, A. (1995). Statistical methods for comparing regression coefficients between models. *American Journal of Sociology, 100*(5), 1261–1293. <https://doi.org/10.1086/230638>). In the table below (now included in an Appendix), the results indicate that out of 741 comparisons, 429 (58%) are statistically different from one another.

And the following to the statistical analysis section (citations in manuscript):

“To investigate whether our state-level RD estimates are statistically different from one another, we conduct a series of z-tests for each state-pair in our sample.”

We have now added the following to the results section:

“Among all state-pairs, our z-tests indicate 58% of regression coefficients were statistically different from one another (Table S6).”

**On the other hand, the regression analyses in Tables 1 and 2 also seem rather weak, primarily because they consist of many bivariate regressions. My understanding is that the authors cannot run multivariate regressions due to the very small sample size, but that is not a good justification of the bivariate analysis (rather, it could indicate that the available data are simply not rich enough to analyze the question of interest satisfactorily).**

The reviewer is correct that small sample sizes limit our ability to full investigate the extent of treatment effect heterogeneity, which we have acknowledged as a limitation in the revised manuscript:

“Lastly, small sample sizes in our bivariable regressions may limit our ability to detect associations with state-level effect estimates. Despite this limitation, we are able to identify associations between our effect estimates and certain important measures such as political affiliation and some demographics. Moreover, the heterogeneity that we find is consistent with prior evidence on heterogeneity in mobility and responsiveness to SIP orders.”

**Moreover, even with such small sample sizes, the authors do find significant relationships between school and business closures and the size of the effect. This suggests that a large part of the between-state variability in the effects of the SIP may be simply capturing the fact that a different bundle of measures was implemented in each state. It could therefore be the case that, if the data were rich enough to account for these differences in policy implementation, the estimated heterogeneity in the effect of SIP could decrease or even disappear. I don't think this problem can be dismissed simply as a minor methodological limitation, as it is serious enough that it undermines the main point of the paper (namely, that the effect of the SIP orders on mobility varied by state).**

The reviewer is correct that SIP implementation varied across states. Unfortunately, a deeper dive into the differences across these 39 individual state SIP orders is outside the scope of this article.

However, we also believe that differences in concurrent policy implementation are unlikely to be the primary driver of this heterogeneity. Indeed, nearly all states in our sample (38 and 32, respectively) implemented the policies that have a relationship with our estimated coefficients (school closures and business closures). Moreover, even among states with every other policy implemented, there remains substantial variation in effect size (mean: -14.8, SD:11.5). We have additionally included the following in the discussion section:

“While the variation in the effectiveness of SIP orders in reducing mobility is likely to partly be a function of other policies implemented concurrently, this is unlikely to be the driver of the effect heterogeneity that we observe. Indeed, nearly all states in our sample (38 and 32, respectively) implemented the policies that have a relationship with our estimated RD coefficients (school closures and business closures). Moreover, even among states with every policy implemented, there remains substantial variation in effect size (mean: -14.8, SD:11.5) Future research should explore the potential mechanisms underlying these differences in more detail.”

**S6 Table:** Z-statistics for difference in state-level coefficients

|  | **AL** | **AK** | **AZ** | **CA** | **CO** | **DE** | **FL** | **GA** | **HI** | **ID** | **IL** | **IN** | **KS** | **LA** | **ME** | **MD** | **MA** | **MI** | **MN** | **MS** | **MO** | **MT** | **NV** | **NH** | **NJ** | **NM** | **NY** | **NC** | **OH** | **OR** | **PA** | **RI** | **SC** | **TN** | **VT** | **VA** | **WA** | **WV** |
| --- | --- | --- | --- | --- | --- | --- | --- | --- | --- | --- | --- | --- | --- | --- | --- | --- | --- | --- | --- | --- | --- | --- | --- | --- | --- | --- | --- | --- | --- | --- | --- | --- | --- | --- | --- | --- | --- | --- |
| **AL** | 0.0 | 1.3 | 2.4 | 3.0 | 1.0 | 0.7 | 0.1 | 4.5 | 0.4 | 0.4 | 1.9 | 3.1 | 4.2 | 3.3 | 0.5 | 3.2 | 2.3 | 0.8 | 3.3 | 5.6 | 0.3 | 2.6 | 3.7 | 3.2 | 0.7 | 2.2 | 6.7 | 2.2 | 0.4 | 1.7 | 5.9 | 2.0 | 6.3 | 11.3 | 2.6 | 1.0 | 1.1 | 0.2 |
| **AK** |  | 0.0 | 0.9 | 0.4 | 1.7 | 0.7 | 1.2 | 0.5 | 0.7 | 1.3 | 2.1 | 0.1 | 1.0 | 0.2 | 1.0 | 0.4 | 2.7 | 0.8 | 2.9 | 1.7 | 1.1 | 0.7 | 1.6 | 1.0 | 0.7 | 0.3 | 4.8 | 2.2 | 1.4 | 2.2 | 1.4 | 2.4 | 1.7 | 3.7 | 0.4 | 0.8 | 1.8 | 1.2 |
| **AZ** |  |  | 0.0 | 0.8 | 2.7 | 1.7 | 2.3 | 0.7 | 1.6 | 2.3 | 3.3 | 1.3 | 0.1 | 1.0 | 2.2 | 0.8 | 3.6 | 1.9 | 4.0 | 0.5 | 2.3 | 0.3 | 0.7 | 0.1 | 1.8 | 0.8 | 5.8 | 3.4 | 2.5 | 3.2 | 0.2 | 3.0 | 0.5 | 2.5 | 0.7 | 2.0 | 2.8 | 2.3 |
| **CA** |  |  |  | 0.0 | 2.9 | 1.4 | 2.5 | 0.2 | 1.3 | 2.3 | 4.4 | 0.9 | 1.1 | 0.4 | 2.5 | 0.0 | 3.7 | 1.9 | 5.4 | 2.2 | 2.5 | 0.5 | 1.8 | 0.9 | 1.7 | 0.1 | 8.2 | 4.6 | 3.0 | 3.6 | 1.8 | 2.8 | 2.3 | 5.9 | 0.1 | 2.1 | 3.0 | 2.7 |
| **CO** |  |  |  |  | 0.0 | 1.3 | 0.9 | 3.5 | 1.0 | 0.4 | 0.2 | 2.7 | 3.8 | 2.9 | 1.3 | 3.0 | 1.4 | 1.4 | 1.2 | 4.7 | 1.2 | 2.8 | 3.8 | 3.3 | 1.4 | 2.5 | 3.7 | 0.3 | 0.7 | 0.6 | 4.6 | 1.6 | 4.9 | 7.7 | 2.7 | 1.6 | 0.1 | 1.1 |
| **DE** |  |  |  |  |  | 0.0 | 0.6 | 1.7 | 0.1 | 0.8 | 1.8 | 0.9 | 2.2 | 1.2 | 0.4 | 1.4 | 2.4 | 0.1 | 2.8 | 3.1 | 0.5 | 1.6 | 2.6 | 2.0 | 0.1 | 1.2 | 5.2 | 1.9 | 0.9 | 1.9 | 2.9 | 2.2 | 3.2 | 5.9 | 1.3 | 0.1 | 1.3 | 0.5 |
| **FL** |  |  |  |  |  |  | 0.0 | 3.3 | 0.4 | 0.4 | 1.6 | 2.2 | 3.5 | 2.5 | 0.3 | 2.6 | 2.3 | 0.6 | 2.8 | 4.7 | 0.2 | 2.3 | 3.4 | 2.9 | 0.6 | 2.0 | 5.8 | 1.8 | 0.4 | 1.6 | 4.7 | 2.0 | 5.1 | 8.8 | 2.2 | 0.7 | 1.0 | 0.1 |
| **GA** |  |  |  |  |  |  |  | 0.0 | 1.6 | 2.8 | 6.3 | 1.8 | 1.1 | 0.8 | 3.7 | 0.3 | 4.1 | 2.6 | 7.1 | 2.5 | 3.6 | 0.4 | 1.8 | 0.8 | 2.2 | 0.3 | 10.3 | 6.5 | 4.1 | 4.2 | 2.2 | 2.9 | 2.8 | 7.9 | 0.1 | 3.1 | 3.5 | 4.0 |
| **HI** |  |  |  |  |  |  |  |  | 0.0 | 0.6 | 1.3 | 0.9 | 2.0 | 1.2 | 0.2 | 1.3 | 2.1 | 0.1 | 2.2 | 2.7 | 0.2 | 1.5 | 2.5 | 1.8 | 0.1 | 1.1 | 4.3 | 1.5 | 0.6 | 1.5 | 2.5 | 2.0 | 2.8 | 5.0 | 1.3 | 0.1 | 1.1 | 0.3 |
| **ID** |  |  |  |  |  |  |  |  |  | 0.0 | 0.7 | 2.0 | 3.1 | 2.3 | 0.7 | 2.4 | 1.7 | 0.9 | 1.7 | 4.0 | 0.6 | 2.3 | 3.3 | 2.8 | 0.8 | 2.0 | 4.1 | 0.9 | 0.1 | 1.0 | 3.8 | 1.8 | 4.2 | 6.8 | 2.2 | 1.0 | 0.5 | 0.5 |
| **IL** |  |  |  |  |  |  |  |  |  |  | 0.0 | 5.0 | 5.6 | 5.0 | 2.2 | 4.7 | 1.5 | 2.3 | 1.6 | 7.0 | 2.0 | 3.6 | 4.6 | 4.3 | 2.0 | 3.4 | 5.1 | 0.3 | 1.3 | 0.6 | 7.5 | 1.6 | 7.8 | 12.7 | 3.8 | 2.7 | 0.1 | 2.0 |
| **IN** |  |  |  |  |  |  |  |  |  |  |  | 0.0 | 2.3 | 0.7 | 2.3 | 1.0 | 3.5 | 1.4 | 6.1 | 3.8 | 2.3 | 1.2 | 2.5 | 1.7 | 1.2 | 0.6 | 9.4 | 5.3 | 2.9 | 3.4 | 3.8 | 2.6 | 4.3 | 9.8 | 0.9 | 1.7 | 2.7 | 2.6 |
| **KS** |  |  |  |  |  |  |  |  |  |  |  |  | 0.0 | 1.6 | 3.7 | 1.1 | 4.4 | 2.9 | 6.5 | 1.1 | 3.7 | 0.2 | 1.0 | 0.0 | 2.6 | 1.0 | 9.2 | 5.8 | 4.1 | 4.4 | 0.6 | 3.2 | 1.1 | 4.6 | 0.9 | 3.3 | 3.8 | 3.9 |
| **LA** |  |  |  |  |  |  |  |  |  |  |  |  |  | 0.0 | 2.6 | 0.4 | 3.7 | 1.8 | 6.0 | 2.9 | 2.7 | 0.8 | 2.1 | 1.3 | 1.6 | 0.2 | 9.1 | 5.2 | 3.1 | 3.6 | 2.7 | 2.7 | 3.3 | 7.9 | 0.4 | 2.1 | 3.0 | 2.9 |
| **ME** |  |  |  |  |  |  |  |  |  |  |  |  |  |  | 0.0 | 2.6 | 2.5 | 0.4 | 3.6 | 5.0 | 0.1 | 2.3 | 3.4 | 2.9 | 0.4 | 1.9 | 6.8 | 2.5 | 0.7 | 2.0 | 5.1 | 2.1 | 5.6 | 10.2 | 2.2 | 0.5 | 1.3 | 0.3 |
| **MD** |  |  |  |  |  |  |  |  |  |  |  |  |  |  |  | 0.0 | 3.8 | 1.9 | 5.7 | 2.3 | 2.7 | 0.6 | 1.8 | 0.9 | 1.7 | 0.1 | 8.5 | 4.9 | 3.1 | 3.7 | 1.9 | 2.8 | 2.5 | 6.3 | 0.1 | 2.2 | 3.0 | 2.9 |
| **MA** |  |  |  |  |  |  |  |  |  |  |  |  |  |  |  |  | 0.0 | 2.6 | 0.7 | 5.0 | 2.4 | 3.6 | 4.4 | 4.0 | 2.5 | 3.4 | 1.3 | 1.4 | 2.1 | 0.9 | 4.9 | 0.7 | 5.2 | 7.1 | 3.6 | 2.7 | 1.4 | 2.4 |
| **MI** |  |  |  |  |  |  |  |  |  |  |  |  |  |  |  |  |  | 0.0 | 3.5 | 4.1 | 0.5 | 1.9 | 3.0 | 2.4 | 0.0 | 1.5 | 6.4 | 2.5 | 1.0 | 2.1 | 4.0 | 2.2 | 4.4 | 8.2 | 1.7 | 0.0 | 1.5 | 0.6 |
| **MN** |  |  |  |  |  |  |  |  |  |  |  |  |  |  |  |  |  |  | 0.0 | 7.8 | 3.3 | 4.4 | 5.3 | 5.2 | 3.1 | 4.3 | 3.3 | 1.4 | 2.6 | 0.5 | 8.2 | 1.1 | 8.5 | 12.8 | 4.7 | 3.9 | 1.2 | 3.3 |
| **MS** |  |  |  |  |  |  |  |  |  |  |  |  |  |  |  |  |  |  |  | 0.0 | 5.0 | 1.0 | 0.3 | 0.8 | 3.7 | 1.9 | 10.4 | 7.2 | 5.3 | 5.3 | 0.6 | 3.5 | 0.0 | 3.5 | 1.8 | 4.6 | 4.7 | 5.3 |
| **MO** |  |  |  |  |  |  |  |  |  |  |  |  |  |  |  |  |  |  |  |  | 0.0 | 2.3 | 3.4 | 2.9 | 0.5 | 2.0 | 6.5 | 2.3 | 0.6 | 1.9 | 5.1 | 2.1 | 5.5 | 9.9 | 2.2 | 0.6 | 1.2 | 0.1 |
| **MT** |  |  |  |  |  |  |  |  |  |  |  |  |  |  |  |  |  |  |  |  |  | 0.0 | 1.1 | 0.2 | 1.8 | 0.5 | 6.5 | 3.7 | 2.7 | 3.3 | 0.7 | 2.9 | 1.0 | 3.4 | 0.4 | 2.0 | 2.8 | 2.4 |
| **NV** |  |  |  |  |  |  |  |  |  |  |  |  |  |  |  |  |  |  |  |  |  |  | 0.0 | 0.9 | 2.9 | 1.7 | 7.3 | 4.7 | 3.7 | 4.2 | 0.7 | 3.5 | 0.4 | 1.7 | 1.6 | 3.1 | 3.8 | 3.5 |
| **NH** |  |  |  |  |  |  |  |  |  |  |  |  |  |  |  |  |  |  |  |  |  |  |  | 0.0 | 2.2 | 0.9 | 7.5 | 4.5 | 3.3 | 3.8 | 0.4 | 3.1 | 0.8 | 3.5 | 0.8 | 2.6 | 3.3 | 3.1 |
| **NJ** |  |  |  |  |  |  |  |  |  |  |  |  |  |  |  |  |  |  |  |  |  |  |  |  | 0.0 | 1.4 | 5.9 | 2.2 | 0.9 | 2.0 | 3.5 | 2.2 | 3.9 | 7.1 | 1.6 | 0.0 | 1.4 | 0.6 |
| **NM** |  |  |  |  |  |  |  |  |  |  |  |  |  |  |  |  |  |  |  |  |  |  |  |  |  | 0.0 | 6.8 | 3.6 | 2.3 | 3.1 | 1.5 | 2.7 | 2.0 | 4.7 | 0.1 | 1.6 | 2.5 | 2.1 |
| **NY** |  |  |  |  |  |  |  |  |  |  |  |  |  |  |  |  |  |  |  |  |  |  |  |  |  |  | 0.0 | 4.8 | 5.8 | 3.0 | 11.1 | 0.1 | 11.3 | 15.3 | 7.3 | 7.1 | 3.6 | 6.6 |
| **NC** |  |  |  |  |  |  |  |  |  |  |  |  |  |  |  |  |  |  |  |  |  |  |  |  |  |  |  | 0.0 | 1.5 | 0.4 | 7.7 | 1.5 | 8.0 | 12.9 | 4.0 | 2.9 | 0.3 | 2.2 |
| **OH** |  |  |  |  |  |  |  |  |  |  |  |  |  |  |  |  |  |  |  |  |  |  |  |  |  |  |  |  | 0.0 | 1.4 | 5.4 | 1.9 | 5.8 | 10.0 | 2.7 | 1.2 | 0.8 | 0.5 |
| **OR** |  |  |  |  |  |  |  |  |  |  |  |  |  |  |  |  |  |  |  |  |  |  |  |  |  |  |  |  |  | 0.0 | 5.2 | 1.3 | 5.6 | 8.3 | 3.3 | 2.3 | 0.5 | 1.8 |
| **PA** |  |  |  |  |  |  |  |  |  |  |  |  |  |  |  |  |  |  |  |  |  |  |  |  |  |  |  |  |  |  | 0.0 | 3.4 | 0.6 | 4.9 | 1.5 | 4.6 | 4.6 | 5.5 |
| **RI** |  |  |  |  |  |  |  |  |  |  |  |  |  |  |  |  |  |  |  |  |  |  |  |  |  |  |  |  |  |  |  | 0.0 | 3.5 | 4.5 | 2.8 | 2.2 | 1.5 | 2.1 |
| **SC** |  |  |  |  |  |  |  |  |  |  |  |  |  |  |  |  |  |  |  |  |  |  |  |  |  |  |  |  |  |  |  |  | 0.0 | 4.0 | 1.9 | 5.1 | 4.9 | 5.9 |
| **TN** |  |  |  |  |  |  |  |  |  |  |  |  |  |  |  |  |  |  |  |  |  |  |  |  |  |  |  |  |  |  |  |  |  | 0.0 | 4.9 | 9.5 | 7.7 | 10.5 |
| **VT** |  |  |  |  |  |  |  |  |  |  |  |  |  |  |  |  |  |  |  |  |  |  |  |  |  |  |  |  |  |  |  |  |  |  | 0.0 | 1.8 | 2.8 | 2.4 |
| **VA** |  |  |  |  |  |  |  |  |  |  |  |  |  |  |  |  |  |  |  |  |  |  |  |  |  |  |  |  |  |  |  |  |  |  |  | 0.0 | 1.6 | 0.7 |
| **WA** |  |  |  |  |  |  |  |  |  |  |  |  |  |  |  |  |  |  |  |  |  |  |  |  |  |  |  |  |  |  |  |  |  |  |  |  | 0.0 | 1.1 |
| **WV** |  |  |  |  |  |  |  |  |  |  |  |  |  |  |  |  |  |  |  |  |  |  |  |  |  |  |  |  |  |  |  |  |  |  |  |  |  | 0.0 |

This table reports z-statistics testing whether each state-pair in our sample has statistically different coefficients. Out of 741 tests, 429 (58%) are statistically different from one another. Green shading indicates a z-statistic greater than 1.96.
